# 小细胞肺癌免疫治疗研究进展

**DOI:** 10.3779/j.issn.1009-3419.2014.06.07

**Published:** 2014-06-20

**Authors:** 菁菁 柳, 爽 张, 慧 李, 颖 程

**Affiliations:** 1 130012 长春，吉林省肿瘤医院胸部肿瘤内科 Department of Thoracic Oncology, Jilin Provincial Cancer Hospital, Changchun 130012, China; 2 130012 长春，吉林省肿瘤医院血液肿瘤病实验室 Hematological Oncology Laboratory, Jilin Provincial Cancer Hospital, Changchun 130012, China

**Keywords:** 小细胞肺癌, 免疫治疗, 肿瘤疫苗, 单克隆抗体, Small cell lung cancer, Immunotherapy, Cancer vaccine, Monoclonal antibody

## Abstract

小细胞肺癌（small cell lung cancer, SCLC）具有复杂的异质性，由于细胞起源、发病机制和驱动基因尚不明确，SCLC的诊治进展缓慢，鲜有突破，迫切需要新的治疗策略提高SCLC疗效。肿瘤免疫治疗可提高免疫系统识别和排除肿瘤细胞的能力，且对正常组织影响轻微。目前已经开展了肿瘤疫苗、过继细胞免疫治疗、细胞因子、checkpoint抑制剂等治疗SCLC的临床研究，ipilimumab是最有前景的药物。免疫治疗有望为SCLC治疗带来新的希望，未来还需要对SCLC的异质性、免疫治疗靶点不明确、免疫治疗耐受等影响免疫治疗疗效的问题开展进一步研究。

## Abstract (en)

Small cell lung cancer (SCLC) is complex heterogeneous due to unclear biological characteristics in terms of cell origin, pathogenesis and driver genes *etc*. Diagnosis and treatment of SCLC has been slowly improved and few breakthroughs have been discovered up to now. Therefore new strategies are urgently needed to improve the efficacy of SCLC treatment. Tumor immunotherapy has potential to restore and trigger the immune system to recognize and eliminate tumor cells, notably it has only minimal adverse impact on normal tissue. Cancer vaccine, adoptive immunotherapy, cytokines and checkpoint inhibitors have now been launched for clinical treatment of SCLC. Ipilimumab is the most promising medicine of immunotherapy. Immunotherapy is expected to bring new vision to the treatment of SCLC. And further researches are needed on such problems affecting efficacy of immunotherapy as the heterogeneity of SCLC, the uncertainty of target for immunotherapy, the immune tolerance, *etc*.

小细胞肺癌（small cell lung cancer, SCLC）是肺癌常见的类型之一，约占肺癌总数的14%，与吸烟明显相关^[1, 2]^。作为一种高侵袭性肿瘤，SCLC疾病进展快，易早期发生转移，由于易复发和产生耐药，中位总生存期仅8个月-11个月，5年生存率不足5%^[3-5]^。由于细胞起源不明、具有复杂的肿瘤异质性、发病机制和驱动基因不明确等原因，SCLC的基础和临床研究发展缓慢，虽然在靶向治疗领域、分子发病机制和全基因组测序等方面进行了探索性研究，但几十年来几乎没有什么突破性进展^[6, 7]^，迫切需要新的研究视角和治疗方法。近年来随着分子生物学、免疫学及生物工程技术在肿瘤学中的研究深入和应用，肿瘤免疫治疗已经成为肿瘤治疗领域的研究热点。免疫治疗不但可提高免疫系统识别和排除肿瘤细胞的能力，且仅对正常组织产生轻微影响，具有广阔的研究和应用前景。研究^[8]^显示，伴有免疫介导的Lambert-Eaton综合征的SCLC患者生存期较长；与复发性SCLC相比，生存期长的SCLC患者其调节性T细胞（regulatory T cell, Treg）水平更高^[9]^，这些结果提示也许可以通过免疫治疗来诱导或强化SCLC的免疫应答，从而达到抗肿瘤的目的。目前开展的SCLC免疫治疗方法主要包括肿瘤疫苗、过继细胞免疫治疗、细胞因子和checkpoint抑制剂等，本文就以上几方面内容介绍SCLC免疫治疗的最新进展。

## 1 肿瘤疫苗研究

肿瘤疫苗的研发是目前肿瘤免疫治疗的热点，肿瘤疫苗通过直接应用肿瘤抗原进行主动免疫治疗，在临床中起到越来越重要的作用。目前认为SCLC免疫治疗可能的靶点在细胞表面上，主要包括神经节苷脂、多唾液酸、糖类抗原Globo H和糖蛋白KSA等。

### 1.1 神经节苷脂肿瘤疫苗

神经节苷脂存在于生长和发育组织，尤其是神经上皮来源的细胞。在许多恶性肿瘤中表达异常，在肿瘤形成、生长、血管生成、转移和肿瘤免疫等诸多过程中起重要作用，也是SCLC最常见的主要分化抗原，其家族包括GM2、GD2、GD3、9-O-acetyl GD3和Fucosyl GM1（Fuc-GM1）等。

#### 1.1.1 Fuc-GM1疫苗

Fuc-GM1在正常肺组织中表达极低，但在SCLC细胞中却表达丰富。既往有研究发现，75%-90%的SCLC肿瘤组织中存在Fuc-GM1的表达，而SCLC血清中大约有50%有Fuc-GM1表达。在培养后的SCLC细胞株中进行免疫染色，肿瘤提取物及鼠的异种种植血清中均有Fuc-GM1表达^[10]^。这种Fuc-GM1对SCLC的特异性提示神经节苷脂抗原可以作为SCLC免疫治疗的靶点，一些Fuc-GM1特异性的单克隆抗体也被用于治疗SCLC的临床研究。最初研究人员^[11]^用牛的甲状腺组织中分离出来的Fuc-GM1与钥孔戚血蓝素（Keyhole Limpet Hemocyanin, KLH）结合形成的共价疫苗治疗了13例SCLC患者，并对10例既往接受过化疗或化放疗且至少完成5次疫苗治疗的患者进行评估，这10例患者在注射Fuc-GM1疫苗后均有血清学缓解，体内抗Fuc-GM1的IgM和IgG抗体水平均升高，提示Fuc-GM1-KLH疫苗对SCLC可产生免疫原性。随后，另外一项研究^[12]^给17例既往接受化疗有效的SCLC患者注射了人工合成的Fuc-GM1-KLH疫苗，患者随机接受30 μg、10 μg、5 μg不同剂量的疫苗。在接受30 μg疫苗的6例患者中，有5例出现抗Fuc-GM1 IgM水平升高，接受10 μg疫苗的5例患者中，有3例IgM水平升高，接受5 μg的患者中没有出现IgM水平的升高。结论认为合成的Fuc-GM1-KLH结合物能够诱导产生针对Fuc-GM1的IgM抗体反应。2009年，Nagorny等^[13]^研发了一种新型Fuc-GM1的疫苗，研究者将主要组织相容性复合体（major histocompatibility complex, MHC）结合位点插入糖分子中，并以选择的序列抗原结合到人HLA-DR的9种不同等位基因，通过一定程度地修饰使免疫原性得到提高，目前正在进行相关的临床研究。

#### 1.1.2 Bec2疫苗

神经节苷脂GD3是细胞表面鞘糖脂，仅在神经外胚层和T淋巴细胞亚型细胞中表达。大部分SCLC肿瘤组织和细胞株中均有神经节苷脂GD3的高表达，而正常组织中GD3的表达水平很低，免疫原性较差。既往一项黑色素瘤研究结果提示神经节苷脂GD3可能是GD3阳性肿瘤免疫治疗适当的抗原靶点^[14]^，抗独特型IgG2b鼠源性单克隆抗体Bec2的结构域与GD3相似，在黑色素瘤患者中表现出很强的免疫原性^[15]^。基于以上研究，研究者们开展了Bec2治疗SCLC的临床研究，同时给予免疫佐剂BCG诱导抗GD3免疫反应。

在第一项Bec2/BCG疫苗治疗15例SCLC患者的临床研究^[16]^中，13例有效病例均产生了抗Bec2的IgM抗体，5例产生抗GD3抗体，3例还产生了IgG抗体，1例患者在治疗结束1年后仍可检测到抗体反应。诊断后的平均生存时间为20.5个月，可检测到GD3抗体的患者疾病复发间隔比其他患者长。广泛期患者的中位无复发生存时间为11个月，7例局限期患者没有达到中位无复发时间，仅有1例患者在随访47个月时出现复发。最严重不良反应为局部皮肤的刺激反应。与接受传统治疗的SCLC患者比较，虽然统计学上无差异，但接受Bec2/BCG疫苗治疗的患者生存期更长。基于该研究结果，研究者们开展了Bec2/BCG疫苗治疗515例化放疗有效的局限期SCLC患者的Ⅲ期临床试验（Silva研究），患者随机接受Bec2（2.5 mg）/BCG疫苗或随访，疫苗组患者在10周内接受5次疫苗注射。主要毒性为注射部位皮肤轻度溃疡和轻度流感样症状。遗憾的是，Bec2/BCG疫苗并不能改善患者的OS和PFS，从随机时间开始计算，观察组和疫苗组的中位生存时间分别为16.4个月和14.3个月（*P*=0.28）^[17]^。生活质量分析数据显示Bec2/BCG疫苗对SCLC患者的生活质量也没有明显改善^[18]^。该研究是全球第一项神经节苷脂疫苗辅助治疗SCLC的Ⅲ期临床试验，只有1/3的患者出现体液免疫应答可能是试验失败的主要原因，另外由于没有对患者组织中GD3表达进行检测，而既往研究显示大概有66%的SCLC组织中存在GD3的阳性表达，所以部分患者无GD3表达也可能是导致阴性结果的原因之一。SCLC肿瘤组织中并不仅仅表达一种神经节苷脂抗原，其他抗原比如GD2等在SCLC中也有较高的表达，是否有其他方法可以改变疫苗治疗策略？比如通过研发多价疫苗的形式增加更多的抗原来是否可提高治疗SCLC的作用尚无定论。目前一种由GD2L、GD3L、Globo H、Fuc-GM1和N-丙酰化多唾液酸（N-Propionylated Polysialic Acid）与偶联物KLH和OPT-821共同组成的五价疫苗正在进行治疗SCLC的临床研究（NCT01349647），将会解答我们的疑问。

### 1.2 Polysialic acid（polySA）疫苗

PolySA存在于革兰氏阴性菌、胚胎样神经冠细胞和一些神经冠起源的恶性细胞表面，可抑制细胞粘附分子的结合，这些特性使其在神经冠细胞迁移和恶性细胞的早期转移过程中发挥重要作用。研究者^[19-21]^采用免疫染色法在几乎所有的SCLC肿瘤中均检测到PolySA的表达，并且与类癌比较，PolySA在SCLC中的表达更明显，所以PolySA在SCLC的疾病进展中可能发挥作用。基于PolySA在正常组织中不表达却在SCLC中表达丰富的特性，PolySA被认为是合理的、高特异性的免疫治疗靶点。

在Krug等^[22]^尝试用PolySA疫苗治疗既往接受初始治疗缓解的SCLC患者的研究中，研究者对患者的免疫原性进行了检测。11例患者接受了两种形式的PolySA疫苗，其中5例患者接受30 μg未修饰的PolySA疫苗，6例接受30 μg与KLH共轭结合的N-丙酰化PolySA疫苗（NP-PolySA-KLH），并且在第1、2、3、4、8和16周时给予100 μg免疫佐剂QS-21。5例接受未修饰PolySA疫苗的患者中有1例出现IgM免疫应答。6例接受NP-PolySA疫苗的患者均检测到IgM应答，其中5例患者对NP-PolySA疫苗产生IgG应答，但不对未修饰PolySA疫苗产生IgG应答。流式细胞仪检测到SCLC细胞株中有IgM抗体表达。除了PolySA的IgM抗体产生外，未发现其他依赖人类补体裂解的PolySA阳性肿瘤细胞。由此可见，接种NP-PolySA-KLH疫苗可产生持续的高滴度抗体反应。此外，研究中还发现个别患者出现周围神经病变和共济失调，所以Krug等^[23]^又对NP-PolySA疫苗在体内产生抗体的最低有效剂量和安全性进行了进一步探索性研究。18例完成初始治疗且没有进展的SCLC患者接受10 μg（*n*=9）或3 μg（*n*=9）NP-PolySA-KLH疫苗（在第1、2、3、4、8和16周时混合注射100 μg免疫佐剂QS-21）。在接受疫苗治疗前，每组各有1例患者可测出低滴度PolySA抗体。10 μg组中的所有患者均产生抗PolySA的IgM抗体（中位滴度1/1，280 ELISA法），除1例患者外均产生抗NP-PolySA的IgM和IgG抗体（中位滴度1/10，240）。3 μg组抗体反应较低，其中6例患者出现PolySA抗体反应（中位滴度1/160）。在接种疫苗后两组各有6/9和3/9的患者血清中出现SCLC细胞强免疫应答反应。18例患者中有1例患者出现了原因不明的3级自限性共济失调。该研究达到了研究目的，证明了NP-PolySA-KLH疫苗的安全性，且具有较好的免疫原性，可产生持续的高滴度抗体反应，也明确了疫苗的最佳剂量为10 μg，以便于开展进一步的临床研究。

### 1.3 树突状细胞疫苗

p53在细胞周期的调节中起重要作用，大约有≥90%的SCLC患者中存在抑癌基因*p53*突变，而正常组织中p53表达低^[24]^。p53与免疫系统功能和免疫反应有明显相关性，p53可以作为肿瘤相关抗原激发机体免疫反应；此外，p53作为转录因子，可以直接启动免疫细胞中某些基因的表达，调节细胞增殖和功能。应用p53抗原肽或野生型p53将有助于激发抗肿瘤免疫反应，通过腺病毒等载体介导*p53*基因转染并表达于肿瘤细胞有明显的治疗作用，从而为肿瘤的免疫治疗带来新的希望。

在一项重组p53腺病毒转染的树突状细胞疫苗治疗29例广泛期SCLC的研究^[25]^中，57.1%的患者出现明显的p53特异性免疫应答。只有1例患者获得临床客观缓解，但在21例接受过二线化疗的患者中观察到较高的临床缓解率（69.1%）。INGN-225是p53修饰腺病毒介导的树突细胞疫苗，Ⅰ期/Ⅱ期临床研究结果显示INGN-225安全性好，可诱导SCLC产生明显的免疫应答。18例/43例（41.8%）患者中特异性抗p53免疫应答阳性，17例/33例（51.5%）患者中观察到全部的Post-INGN-225应答。阳性和阴性免疫应答的患者中观察到的Post-INGN-225应答病例数分别为11/14（78.6%）和5/15（33%）。产生免疫应答和无免疫应答患者的中位生存期分别为12.6个月和8.2个月（*P*=0.131）。明确诱导免疫应答和化疗-免疫治疗协同作用的机制可能会更好地阐释其抗肿瘤治疗作用^[25]^。目前正在进行一项INGN-225联合化疗药物紫杉醇和全反式维甲酸治疗广泛期SCLC的Ⅱ期临床研究（NCT00617409），以进一步证实INGN-225是否可以增加抗肿瘤活性。

## 2 SCLC的过继性细胞免疫治疗

过继性细胞免疫治疗以自身免疫细胞为基础，向肿瘤患者回输具有抗肿瘤活性的免疫细胞，激发机体免疫反应以杀伤肿瘤细胞^[26]^。过继性细胞免疫治疗被广泛应用于临床试验和实验研究，在肺癌领域开展的研究主要有细胞因子诱导的杀伤细胞（cytokine induced killer cell, CIK cell）、淋巴因子激活的杀伤细胞（lymphokine-activated killer cell, LAK cell）、肿瘤浸润淋巴细胞（tumor-infiltrating lymphocytes cell, TIL cell）和γδ T细胞等，但多为非小细胞肺癌（non-small cell lung cancer, NSCLC）相关研究^[27]^。目前正在进行一项自体CIK细胞维持治疗SCLC的Ⅱ期临床研究，试验组每月给予自体CIK细胞治疗，直至疾病进展；对照组给予最佳支持治疗，以探讨CIK细胞治疗是否能够改善SCLC的生存期和生活质量（NCT01592422）。

## 3 细胞因子

细胞因子是由细胞免疫系统分泌的小分子蛋白，干扰素（interferon, IFN）是重要的细胞因子之一，对抗体产生、自然杀伤细胞（natural killer, NK）和T细胞活性、巨噬细胞功能、迟发型过敏反应和MHC表达等均有一定的免疫调节作用。既往有研究^[28]^报道IFN-α有明显的抗肿瘤活性且可改善SCLC的生存期。IFN-α和IFN-β可抑制肿瘤细胞生长并激活免疫系统，IFN-γ也可以诱导免疫调节和抗肿瘤细胞增殖。IFNs联合化疗治疗SCLC的Ⅱ期随机研究评价了化疗的SCLC患者接受免疫治疗的有效性、生存改善和某些免疫标志物表达情况^[29]^。164例SCLC患者（局限期和广泛期）随机接受化疗（A组）或化疗联合免疫治疗，免疫治疗又分以下三种情况：IFN-α（300万IU）每周3次（B组）；IFN-γ（300万IU）每周3次（C组）；IFN-α和IFN-γ（均为150万IU）每周3次（D组），所有组别的化疗方案均为卡铂5.5 mg/m^2^，异环磷酰胺3.5 mg/m^2^，第1天静注，依托泊苷总剂量200 mg/m^2^，第1-3天口服，每28天为1个周期，共8个周期。患者完成化疗后重新分期，局限期的患者接受原发灶放疗和预防性脑照射，放疗期间和随访期继续给予免疫治疗。每次化疗前和随访期间均要检测血液中CD3^+^淋巴细胞，CD3^+^CD4^+^淋巴细胞，CD3^+^CD8^+^淋巴细胞，NK细胞和NK-T细胞。结果显示，各组患者的缓解率和生存期没有明显差异。然而，在局限期患者中，*Kaplan-Meier*分析显示B组有生存获益（*P* < 0.05）。对免疫指标检测结果分析后发现免疫标志物的改善通常伴随着临床获益，而免疫标志物恶化伴随着疾病进展（结果没有统计学差异，C组除外，*P* < 0.05）。研究认为，只有IFN-α能够给局限期SCLC患者带来获益，但因为随机患者较少，故还需要进一步开展IFN-α作为辅助和/或维持治疗SCLC的研究加以验证。

## 4 Checkpoint抑制剂

免疫豁免假说认为肿瘤组织内可产生免疫抑制分子，逃避免疫系统的监视，肿瘤细胞诱导调节性T细胞，使活化的免疫效应细胞在肿瘤微环境中不能发挥作用^[30]^，阻断免疫系统的负性调节机制是免疫治疗的策略之一。肿瘤细胞逃避机体免疫系统对其识别及清除的机制有很多，目前研究热点是机体免疫抑制信号通路，其涉及的分子包括细胞毒性T淋巴细胞相关抗原4（cytotoxic T-lymphocyte-associated antigen-4, CTLA-4）、程序性死亡因子-1（programmed death 1, PD-1）/PD-L1等，相应的单克隆抗体药物如ipilimumab、nivolumab、BMS-936559等已经应用于肿瘤的免疫治疗并获得了较好的效果。

### 4.1 抗CTLA-4单克隆抗体

CTLA-4是第一个被作为靶点的免疫checkpoint受体，是免疫球蛋白超家族的成员和细胞毒性T淋巴细胞（cytotoxic T lyphocyte, CTLs）表面受体之一，仅在T细胞中表达，是特异性抗肿瘤反应的重要介质，参与免疫反应的负调节。正常情况下，T细胞的激活需要依赖第一信号（抗原-抗体复合物的形成）和第二信号（B7介导的活化信号）的双活化。T细胞活化后，CTLA-4在T细胞表面表达，通过和CD80（B7.1）/CD86（B7.2）结合，抑制B7家族分子与CD28的结合效率，进而影响T细胞的活化，降低细胞毒性T细胞的肿瘤杀伤效力^[31, 32]^。抗CTLA-4单克隆抗体阻断CTLA-4后，解除其与CD80/CD86的抑制功能，提高机体的抗肿瘤免疫应答，消除免疫抑制效应，调动特异性抗肿瘤免疫反应。

Ipilimumab（Yervoy, MDX-010, BMS-734016）是抗CTLA-4的全人源单克隆IgG1κ抗体，属于新型的T细胞增强剂和免疫系统激活剂。早期临床试验^[33-36]^结果显示ipilimumab治疗多种肿瘤中均可得到较好的缓解。两项Ⅲ期研究^[37, 38]^证实ipilimumab是第一个被发现的可延长晚期黑色素瘤患者总生存期的药物，部分难治性晚期黑色素瘤患者可对其产生持久且完全的应答，因此2011年美国食品药品监督管理局批准ipilimumab用于治疗转移性黑色素瘤。目前有10余项在研的临床研究分别考察其治疗NSCLC、SCLC及前列腺癌等的有效性和安全性。

有研究^[9]^发现长期生存的SCLC患者与复发SCLC患者相比，T细胞调节的抗肿瘤效应较高。所以，ipilimumab可能会抑制SCLC肿瘤的生长并成为一种有前景的免疫抗肿瘤药物。研究者们开展了一系列ipilimumab治疗SCLC的临床研究（表 1）。一项随机、双盲、Ⅱ期临床研究（CA184-041）^[39]^评估了ipilimumab治疗肺癌的安全性与疗效，采用紫杉醇/卡铂联合或不联合ipilimumab治疗Ⅲb期/Ⅳ期NSCLC或广泛期SCLC患者。130例初治的广泛期SCLC患者被随机分为以下3组：使用ipilimumab 10 mg/kg，前4周期同步联合紫杉醇/卡铂化疗（同步方案组），在3个-6个周期的紫杉醇/卡铂化疗中使用ipilimumab 10 mg/kg（分阶段组）或单纯的紫杉醇/卡铂化疗（对照组）。紫杉醇/卡铂化疗6个周期后，根据最初的分组，每12个周接受ipilimumab或安慰剂治疗。主要研究终点是免疫相关的PFS（immune-related PFS, irPFS），即采用免疫相关应答标准（immune-related response criteria, irRC）评估PFS。结果显示，ipilimumab组的irPFS明显高于对照组（HR=0.64, *P*=0.03），但没有改善PFS（HR=0.93, *P*=0.37）和OS（HR=0.75, *P*=0.13）。分阶段组、同步方案组和对照组的中位irPFS分别为6.4个月、5.7个月和5.3个月，中位PFS分别为5.2个月、3.9个月和5.2个月，中位OS分别为12.9个月、9.1个月和9.9个月。3级/4级irAEs的发生率分别为17%、21%和9%，分阶段组显示出较好的临床活性。基于以上结果，ipilimumab是一种非常有前景的可提高SCLC生存的免疫靶向药物，目前正在进行ipilimumab联合/不联合化疗治疗SCLC的临床研究（表 1），其中CA184-156研究是目前为止唯一一项ipilimumab治疗SCLC的Ⅲ期研究（NCT10450761），中国也加入到该研究中，将提供更多中国人群应用ipilimumab的临床数据。

**1:** 目前开展的ipilimumab治疗SCLC的Ⅱ期/Ⅲ期临床研究 Summary of phase Ⅱ/Ⅲ clinical trials with ipilimumab in SCLC

Clinical trial identifier	Phase	Treatment arms	Patient population	Primary endpoint	Status
NCT00527735 (CA184-041)	Ⅱ	Ipilimumab or placebo+PC^*^ (concurrent) *vs* ipilimumab or placebo+PC (sequential) *vs* placebo/PC	ES-SCLC^1^ (and NSCLC)	EORTC-QLQ-30 questionnaire	Completed
NCT01450761 (CA184-156)	Ⅲ	Ipilimumab+EP^#^ *vs* EP	ES-SCLC	OS	Ongoing
NCT01331525 (ICE)	Ⅱ	Ipilimumab+Carboplatin/Etoposide	ES-SCLC	1-yr PFS	Ongoing
NCT02046733 (STIMULI)	Ⅱ	Consolidation ipilimumab *vs* Observation	LS-SCLC^△^	OS	Ongoing
^*^Paclitaxel/Carboplatin; ^#^Etoposide/Cisplatin; ^1^Extensive stage small cell lung cancer; ^△^Limited stage small cell lung cancer (SCLC). NSCLC: non-small cell lung cancer.					

### 4.2 抗PD-1/PD-L1单克隆抗体

程序性死亡因子1（programmed death 1, PD-1）是一种Ⅰ型跨膜糖蛋白，属于免疫球蛋白超家族成员，主要表达在活化T细胞上，以单体的形式存在于细胞表面，PD-L1是PD-1最主要的配体，与受体结合后可抑制T细胞的活化和增殖，诱导T细胞凋亡，从而诱导肿瘤细胞的免疫逃逸^[40]^。临床前研究^[41]^发现，阻断PD-1/PD-L1信号可以促进肿瘤抗原特异性T细胞的增殖，增强免疫反应，从而发挥杀伤肿瘤细胞的作用，且PD-L1的表达水平可能与患者的临床及预后紧密相关。抗PD1抗体不仅能解除T细胞抑制，还能消除肿瘤组织中T_Reg_细胞数量，增强NK细胞功能和抗体表达。

Nivolumab（BMS-936558）是一种人源性抗PD-1的IgG4单克隆抗体。Nivolumab治疗包括NSCLC在内的Ⅰ期临床研究^[42]^结果显示，nivolumab能延长晚期NSCLC患者的生存期，并具有很好的安全性，目前正在进行其治疗NSCLC的Ⅲ期临床研究。BMS-936559是一种高亲和性的特异性人源PD-L1 IgG4单克隆抗体。可以抑制PD-L1与PD-1和CD80 T细胞的结合，阻断活化的T细胞表面的PD-1受体，通过抑制PD-1和PD-L1通路挽救耗竭的T细胞，增强抗肿瘤免疫力。BMS-936559治疗包括NSCLC、黑色素瘤、结直肠癌、肾癌等实体瘤在内的Ⅰ期研究结果也显示出较好的疗效，PD-1和PD-L1是否可以作为SCLC潜在的免疫治疗靶点也是值得思考的问题，也期待能开展相关药物治疗SCLC的临床研究。

此外，由于抗CTLA-4抗体和抗PD-1/PD-L1抗体的免疫作用机制不同，联合免疫治疗是否可以获得更好的疗效？评价ipilimumab和nivolumab联合治疗黑色素瘤的Ⅰ期研究结果提示，联合治疗晚期黑色素瘤优于单药，且具有快速的、持久的、治疗性的反应^[43]^。这种免疫靶向药物联合治疗的模式取得了良好疗效，也为SCLC免疫靶向治疗开启了新的治疗模式视角，目前正在进行的一项Ⅰ期/Ⅱ期研究就评价了单药nivolumab或nivolumab联合ipilimumab治疗4种晚期或转移实体瘤（SCLC、三阴乳腺癌、胃癌和胰腺癌）的安全性和有效性（NCT01928394）。希望随着对肿瘤免疫反应分子机制研究的不断深入，发掘更多的免疫治疗靶点，从而研发相关的抗体类靶向药物，为SCLC治疗带来突破。

## 5 结语

近年来，免疫治疗在肿瘤治疗中凸显出其抗肿瘤优越性，根据现有的研究结果，ipilimumab是SCLC免疫治疗最有前景的药物。SCLC的免疫治疗研究尚处于起步阶段，仍面临着许多挑战，如SCLC肿瘤负荷较大、有复杂的肿瘤异质性、特异性的免疫治疗靶点尚未明确、如何克服肿瘤对免疫治疗耐受等。在免疫治疗的临床试验中还发现除了近期疗效外，远期疗效也需要考虑在内，所以用传统的实体瘤疗效评价标准并不能客观反应免疫治疗的疗效，因此癌症研究所癌症免疫治疗联合会目前已提出免疫治疗相关疗效评价分类（irRC）体系来评价疗效^[44]^。随着肿瘤免疫治疗研究的深入，肿瘤抗原、免疫佐剂和递送系统的研究将越发明晰，免疫治疗必将在SCLC的治疗中占有一席之地。SCLC与神经内分泌密切相关，而神经内分泌通路在调节机体免疫系统方面发挥重要作用，对SCLC的神经内分泌干预是否可以调控免疫功能目前还缺乏相关报道，应该广泛开展相关基础和临床研究。此外，还需要明确免疫治疗的优势人群、发掘更多的免疫治疗靶点、免疫治疗相关的预后和预测标志物及免疫治疗的药物剂量、疗程、用药时机、与化放疗联合用药模式、免疫治疗后患者的临床疗效是否与免疫反应有相关性等问题，才能真正开辟SCLC治疗的新领域。
